# Preferential expression of potential markers for cancer stem cells in large cell neuroendocrine carcinoma of the lung. An FFPE proteomic study

**DOI:** 10.1186/2043-9113-1-23

**Published:** 2011-09-03

**Authors:** Masaharu Nomura, Tetsuya Fukuda, Kiyonaga Fujii, Takeshi Kawamura, Hiromasa Tojo, Makoto Kihara, Yasuhiko Bando, Adi F Gazdar, Masahiro Tsuboi, Hisashi Oshiro, Toshitaka Nagao, Tatsuo Ohira, Norihiko Ikeda, Noriko Gotoh, Harubumi Kato, Gyorgy Marko-Varga, Toshihide Nishimura

**Affiliations:** 1 Dept. of Surgery I, Tokyo Medical University, Tokyo, Japan; 2 Diagnostic Pathology, Division, Tokyo Medical University, Tokyo, Japan; 3 Biosys Technologies, Inc., Tokyo, Japan; 4 Dept. of Structural Biology, Graduate School of Pharmaceutical Science. Hokkaido, University, Hokkaido, Japan; 5 Laboratory for Systems Biology and Medicine, RCAST, The University of Tokyo, Tokyo, Japan; 6 Dept. of Biophysics and Biochemistry, Osaka University, Graduate School of Medicine, Suita, Japan; 7 Medical ProteoScope Co., Ltd. Tokyo, Japan; 8 Hamon Center for Therapeutic Cancer Research, UT Southwestern Medical Center, Texas, USA; 9 Division of Systems Biomedical Technology, The Institute of Medical Science, The University of Tokyo, Tokyo, Japan; 10 Niizashiki Central General Hospital, Saitama, Japan; 11 Clinical Protein Science & Imaging, Dept. of Measurement Technology and Industrial Electrical Engineering, Lund University, Lund, Sweden

**Keywords:** large cell neuroendocrine carcinoma, formalin-fixed paraffin embedded tissues, mass spectrometry, cancer stem cell markers

## Abstract

**Background:**

Large cell neuroendocrine carcinoma (LCNEC) of the lung, a subtype of large cell carcinoma (LCC), is characterized by neuroendocrine differentiation that small cell lung carcinoma (SCLC) shares. Pre-therapeutic histological distinction between LCNEC and SCLC has so far been problematic, leading to adverse clinical outcome. We started a project establishing protein targets characteristic of LCNEC with a proteomic method using formalin fixed paraffin-embedded (FFPE) tissues, which will help make diagnosis convincing.

**Methods:**

Cancer cells were collected by laser microdissection from cancer foci in FFPE tissues of LCNEC (*n *= 4), SCLC (*n *= 5), and LCC (*n *= 5) with definite histological diagnosis. Proteins were extracted from the harvested sections, trypsin-digested, and subjected to HPLC/mass spectrometry. Proteins identified by database search were semi-quantified by spectral counting and statistically sorted by pair-wise G-statistics. The results were immunohistochemically verified using a total of 10 cases for each group to confirm proteomic results.

**Results:**

A total of 1981 proteins identified from the three cancer groups were subjected to pair-wise G-test under *p *< 0.05 and specificity of a protein's expression to LCNEC was checked using a 3D plot with the coordinates comprising G-statistic values for every two group comparisons. We identified four protein candidates preferentially expressed in LCNEC compared with SCLC with convincingly low *p*-values: aldehyde dehydrogenase 1 family member A1 (AL1A1) (*p *= 6.1 × 10^-4^), aldo-keto reductase family 1 members C1 (AK1C1) (*p *= 9.6x10^-10^) and C3 (AK1C3) (*p *= 3.9x10^-10^) and CD44 antigen (*p *= 0.021). These *p*-values were confirmed by non-parametric exact inference tests. Interestingly, all these candidates would belong to cancer stem cell markers. Immunohistochmistry supported proteomic results.

**Conclusions:**

These results suggest that candidate biomarkers of LCNEC were related to cancer stem cells and this proteomic approach via FFPE samples was effective to detect them.

## Introduction

Lung cancer is the leading cause of cancer-related death worldwide [1]. In Japan, annual deaths from lung cancer have been increasing and reached about 70,000 [2] and in USA reached 160,000 even with a recent decreasing trend [3]. Generally, lung cancer is divided into two histological subgroups, non-small cell lung carcinoma (NSCLC) and small cell lung carcinoma (SCLC). NSCLC mainly consists of adenocarcinoma (AC), squamous cell carcinoma (SC) and large cell carcinoma (LCC). AC and SC are differentiated with the features of normal cells but LCC is undifferentiated without such features. The prognosis of lung cancer depends on pathological stages and histological types; in NSCLC, AC is the best, while LCC the worst [4].

Travis et al. [5] proposed a new subtype of LCC, named large cell neuroendocrine carcinoma (LCNEC) in 1991, and the World Health Organization finally adopted it for the revised pathological classification of lung cancer in 1999. LCNEC exhibits morphology similar to LCC but neuroendocrine differentiation like SCLC that could be judged by expression of at least one of three representative neuroendocrine proteins, CD56, synaptophysin (Syn) and chromogranin A (CGA). Among subtypes of LCC, the prognosis of LCNEC was poorer than others even if at early stages [6,7] like SCLC. However therapeutic strategies of LCNEC and SCLC differ from each other. The former needs surgery as the first choice but the latter chemotherapy. It is therefore important to distinguish LCNEC from SCLC definitely but common morphological growth patterns characteristic of neuroendocrine tumors sometimes hinder clear pathologic distinction between the two neuroendocrine cancers.

It follows that new biomarkers should be developed for definite diagnosis of those cancers, even if histopathology has long been the golden standard for diagnosis and determination of disease progression. Genomic and immunohistochemical analyses for such a purpose have been reported [8,9] but there have still been no biomarkers specific to LCNEC. Recent advancements in shotgun sequencing and quantitative mass spectrometry for protein analyses could make proteomics amenable to clinical biomarker discovery [10]. In addition, selective collection of target cells from formalin fixed paraffin embedded (FFPE) tissues by laser microdissection can permit to access to tissues of a variety of cancer types with definite diagnosis. We have used these methods for exploring stage-related proteins on non-metastatic lung AC by both global and multiple reaction monitoring (MRM) mass spectrometry-based proteomics [11,12]. In this study, we applied them to detect the potential protein markers characteristic of LCNEC by label-free semi-quantitative shotgun proteomics using spectral counting.

## 2. Materials and methods

### 2. 1. Sample Preparation for FFPE Tissue Specimens

Surgically removed lung tissues were fixed with a buffered formalin solution containing 10-15% methanol, and embedded by a conventional method. Archived paraffin blocks of formalin-fixed tissues obtained from four LCNEC cases, five LCC and five SCLC, which were retrieved with the approval from Ethical Committee of Tokyo Medical University Hospital and used with patients' consents. Patients' characteristics are listed in Table 1. Paraffin blocks were cut into 4 μm sections for diagnosis and 10 μm sections for proteomics. The 10 μm sections were stained with only haematoxylin. Three pathologists (M.N., H.O., and T.N.) independently made a diagnosis using the 4 μm sections stained with haematoxylin and eosin according to the WHO classification. LCNEC has its characteristic cancer cells with relatively larger cytoplasm, less fine chromatin and more distinct nucleoli than those of SCLC. The sections of patients diagnosed unequivocally were used in this study.

**Table 1:** Patients' Characteristics.

Cancer groups	**Patient No**.	Gender	Age	TNM*	Staging
LCNEC	1	F	68	T1N0M0	IA
	2	M	73	T2N0M0	IB
	3	M	58	T1N1M0	IIA
	4	M	70	T2N0M0	IB
	
	5	M	76	T2N2M0	IIIA
	6	M	69	T3N3M0	IIIB
	7	M	64	T2N1M0	IIB
	8	M	60	T2N2M0	IIIA
	9	F	77	T1N0M0	IA
	10	M	69	T1N2M0	IIIA

SCLC	1	F	62	T2N0M0	IB
	2	M	77	T2N1M0	IIB
	3	M	57	T2N1M0	IIB
	4	M	76	T1N1M0	IIA
	5	M	64	T1N1M0	IIA
	
	6	F	70	T1N1M0	IIA
	7	M	69	T1N1M0	IIA
	8	M	77	T2N0M0	IB
	9	M	73	T1N0M0	IA
	10	M	73	T2N1M0	IIB

LCC	1	M	52	T2N1M0	IIB
	2	M	71	T1N0M0	IA
	3	F	57	T1N0M0	IA
	4	M	51	T4N2M0	IIIB
	5	M	72	T1N1M0	IIA
	
	6	M	67	T1N1M0	IIA
	7	M	67	T2N0M0	IB
	8	M	58	T1N0M0	IA
	9	M	67	T2N0M0	IB
	10	M	66	T1N0M0	IA

*Ref. [31]

### 2. 2. Immunohistochemical Staining

The neuroendocrine nature of tumors was confirmed with the three representative antibodies, monoclonal mouse anti CD56 antibody (Novocastra, Newcastle upon Tyne, U.K.), polyclonal rabbit anti CGA antibody (DAKO Japan, Kyoto, Japan) and monoclonal mouse anti SYN antibody (DAKO Japan, Kyoto, Japan). The staining of these antibodies was performed automatically on a Ventana Benchmark^® ^XT (Ventana Japan, Tokyo, Japan). Expression of four proteomics-identifying proteins specific to LCNEC was tested with the following commercially available antibodies according to the manufacturer's protocols: monoclonal rabbit anti AL1A1 antibody (Abcom Japan, Tokyo, Japan), polyclonal anti AK1C1 antibody (GeneTex, Irvine, CA, USA), monoclonal anti AK1C3 antibody (Sigma Japan, Tokyo, Japan) and monoclonal mouse anti CD44 antibody (Abcom Japan, Tokyo, Japan). Briefly, sections were incubated with xylene, rehydrated with graded ethanol solutions and incubated with methyl alcohol containing 3% hydrogen peroxide to remove endogenous peroxidase activity. After washing thoroughly with PBS, sections were incubated with adequately diluted primary antibodies and then with Histofine simple stain^® ^(Nichirei Bioscience, Tokyo, Japan), and finally visualized with products of the peroxidase and diaminobenzidien reaction.

### 2. 3. Laser Capture and Protein Solubilization

Cancerous lesions were identified on serial sections of NSCLC tissues stained with hematoxylin-eosin (HE). For proteomic analysis, a 10 μm thick section prepared from the same tissue block was attached onto DIRECTOR™ slides (Expression Pathology, Rockville, MD, USA), de-paraffinized twice with xylene for 5 min., rehydrated with graded ethanol solutions and distilled water and stained by only hematoxylin. Those slides were air-dried and subjected to laser microdissection with a Leica LMD6000 (Leica Micro-systems GmbH, Ernst-Leitz-Strasse, Wetzlar, Germany). At least 30,000 cells (8.0mm^2^) were collected directly into a 1.5mL low-binding plastic tube. Proteins were extracted and digested with trypsin using Liquid Tissue™ MS Protein Prep kits (Expression Pathology, Rockville, MD, USA) according to the manufacturer's protocol.

### 2. 4. Liquid Chromatography-Tandem Mass Spectrometry

We here adopted label-free semi-quantitation using spectral counting by liquid chromatography (LC)-tandem mass spectrometry (MS/MS) to a global proteomic analysis. The digested samples were analyzed in triplicates by LC-MS/MS using reversed-phase liquid chromatography (RP-LC) interfaced with a LTQ-Orbitrap hybrid mass spectrometer (Thermo Fisher Scientific, Bremen, Germany) via a *nano*-electrospray device as described in details previously [13]. Briefly, the RP-LC system consisted of a peptide Cap-Trap cartridge (0.5 × 2.0 mm) and a capillary separation column (an L-column Micro of 0.2 × 150 mm packed with reverse phase L-C18 gels of 3 μm in diameter and 12 nm pore size, (CERI, Tokyo, Japan)) connected an emitter tip (FortisTip of 20 μm ID and 150 μm OD with a perfluoropolymer-coated blunt end, OmniSeparo-TJ, Hyogo, Japan) to the outlet. An autosampler (HTC-PAL, CTC Analytics, Switzerland) loaded an aliquot of samples onto the trap, which then was washed with solvent A (98% distilled water with 2% acetonitrile and 0.1% formic acid) for concentrating peptides on the trap and desalting. Subsequently, the trap was connected in series to the separation column, and the whole columns were developed for 70 min. with a linear acetonitrile concentration gradient made from 5 to 40% solvent B (10% distilled water and 90% acetonitrile containing 0.1% formic acid) at the flow-rate of 1 μL/min. An LTQ was operated in the data-dependent MS/MS mode to automatically acquire up to three successive MS/MS scans in the centroid mode. The three most intense precursor ions for these MS/MS scans could be selected from a high-resolution MS spectrum (a survey scan) that an Orbitrap previously acquired during a predefined short time window in the profile mode at the resolution of 30 000 in the *m/z *range of 400 to 1600. The sets of acquired high-resolution MS and MS/MS spectra for peptides were converted to single data files and they were merged into Mascot generic format files for database searching.

### 2.5 Database Searching and Semi-quantification with Spectral Counting

Mascot software (version 2.1.1, Matrix Science, London, UK) was used for database search against Homo sapiens entries in the UniProtKB/Swiss-Prot database (Release 56.6, 20413 entries). Peptide mass tolerance was 10ppm, fragment mass tolerance 0.8Da, and up to two missed cleavages were allowed for errors in trypsin specificity. Carbamidomethylation of cysteines was taken as fixed modifications, and methionine oxidation and formylation of lysine, arginine and N-terminal amino acids as variable modifications. A *p*-value being < 0.05 was considered significant, and the score cutoff was 44. The lists of identified proteins were merged into a master file where the primary accession numbers and entry names from UniProtKB were used. The false positive rates for protein identification were estimated using a decoy database created by reversing the protein sequences in the original database; the estimated false positive rate of peptide matches was 0.45% under protein score threshold conditions (*p *< 0.005). Mascot search results were processed through Scaffold software (version 2.02.03, Proteome Software, Portland, OR) to semi-quantitatively analyze differential expression levels of proteins in LCNEC, LCC and SCLC by spectral counting as described [11]. The number of peptide MS/MS spectra with high confidence (Mascot ion score, p < 0.005) was used for calculating spectral counts. Fold changes of expressed proteins in the base 2 logarithmic scale (*R_SC_*) were calculated using spectral counting as described [11]. Candidate proteins between two groups were chosen so that their *R_SC _*satisfy >1 or <−1, which correspond to their fold changes >2 or <0.5. G-test was used for evaluating differential protein expression in pair-wise cancer groups [14]. In this study we mainly focus on LCNEC vs. SCLC comparison, but the other pairs were considered. The results are illustrated in a three-dimensional plot to judge whether a protein is specifically expressed in a given cancer group. Although G-test does not require replicates, spectral counts for each protein from triplicates were pooled and used for G-statistic calculation using a two-way contingency table arranged in two rows for a target protein and any other proteins, and two columns for cancer groups on an Excel macro. Statistical significance should be *p *< 0.05. The Yates correction for continuity is applied to the 2 × 2 tables. The correction could enable us to handle the data containing small spectral counts including zero. Statisticians, however, showed that the results of G-test using a contingency table containing small counts are not so convincing because it is assumed that the G statistic asymptotically obey a χ^2 ^distribution with one degree of freedom. To validate the G-test results, we calculated exact *p*-values for some significant proteins without making any assumptions of statistical distribution based on the permutational distribution of the test statistic, i.e., Fisher's exact test and Mann-Whitney U test for the contingency tables using a R package.

## 3. Results

### 3. 1. Patient groups and pathological classification

**Figure 1:**
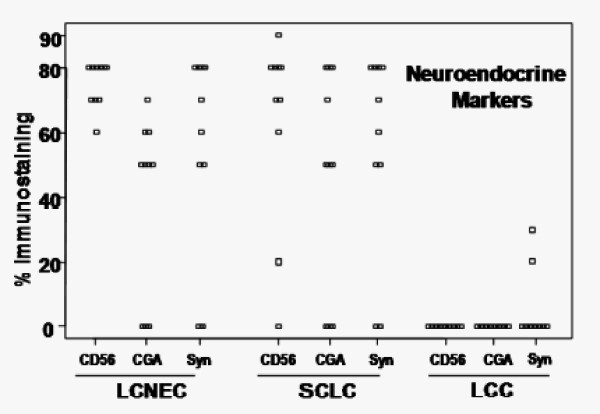
**Immunohistochemistry with antibodies raised against established neuroendocrine markers, CD56, CGA, and Syn**.

To explore protein markers to distinguish LCNEC from SCLC, we investigated cancer cells prepared by laser microdissection from FFPE sections of LCNEC, SCLC, and LCC with a shotgun proteomic method. The LCNEC group consisted of four independent patients and other two groups consisted of five independent ones. For immunohistochemistry, we added more patients so as to amount to 10 patients for each group. Patients were divided into those cancer groups according to the WHO classification and by immunohistochemistry with antibodies raised against established neuroendocrine markers, CD56, CGA and Syn (Table 1 and Figure 1). All LCNEC and SCLC tissues used in this study are positively stained with at least one of these antibodies consistent with the neuroendocrine nature of those cancers. LCC tissues were not stained immunohistochemically except for 2 cases with faintly positive for Syn but histopathological differentiation from SC, AC and SCLC was required for its definite diagnosis. The patient profiles including the TNM pathological classification and staging are summarized in Table 1. There was no difference between the ages for each group (*p *= 0.076 by ANOVA, mean age + SD: 68.4 + 6.3 for LCNEC, 69.8 + 6.8 for SCLC, and 62.8 + 7.7 for LCC) and the number of male accounts for over 80% for all groups. The majority of patients remained at stages from IA to IIB and accordingly had the extent of the primary tumor (T1 and T2) and of regional lymph node involvement (N0 and N1) except for the most advanced stage IIIA or IIIB in a LCC patient (patient 4) and additional four patients of LCNEC for immunohistochemistry (patients 5, 6, 8, and 10). All patients had no distant metastasis (M0). All the patients but patient 5 (carboplatin + irinotecan) in LCNEC and patient 4 (carboplatin + pacritaxel) in LCC have not undergone pre-operative chemotherapy.

### 3. 2. LC-MS/MS protein identifications and semi-quantification by spectral counting

Trypsin-digests from laser-microdissected samples typically containing ~30,000 cells were analyzed in triplicate by LC-MS/MS as described in "Materials and Methods". Under the database search settings used, we identified significant proteins as follows: LCNEC contained a total of 1,124 proteins including 410 unique, 168 in the overlap only between LCNEC and SCLC, 93 in the overlap only between LCNEC and LCC, and 453 in the overlap among three groups; SCLC contained a total of 1,096 including 362 unique, 100 in the overlap only between SCLC and LCC and the overlapped proteins described above; LCC contained a total of 1,083 including 450 unique and the overlapped proteins described earlier. The spectral counts were calculated for these proteins and those from triplicate experiments were pooled, thereby improving the performance of G-test and decreasing false positive rates significantly [14]. There was no significant difference among the total spectral counts of each group (*p *= 0.248 by ANOVA; mean counts + SD: 1916 + 571 for LCNEC, 1879 + 457 for SCLC, 2491 + 645 for LCC). Next, the values of *R*_sc _that is a measure of fold changes for protein expression levels were calculated as described in "Materials and Methods" using the spectral counts of these proteins. The pooled counts for each protein were also subjected to pair-wise G-test between cancer groups. Table 2 shows the identified proteins that are significantly up- or down-regulated in LCNEC compared with SCLC as judged by G test under *p *<0.05. The proteins are listed in descending order of the *R*_sc _values; the larger the *R*_sc _value of a given protein, the greater its expression level in LCNEC compared with SCLC and vice versa. Representative proteins up-regulated in LCNEC were AL1A1, AK1C1, AK1C3, brain-type fatty acid-binding protein (FABP) and β-enolase. On the other hand, those in SCLC were brain acid soluble protein 1 (BASP), secretagogin (SEGN), fascin and neural cell adhesion molecule (CD56).

**Table 2:** Significant changes in protein expression levels as judged with G-test under *p *< 0.05 for an LCNEC vs. SCLC pair.

No	Entry name	Accession number	Proteins	*G*	*P*	*R*sc	Spectral counts	
							
							LCNEC SCLC	
1	AK1C3	P42330	Aldo-keto reductase family 1 member C3	39.1	3.93E-10	4.91	25	0
2	AK1C1	Q04828	Aldo-keto reductase family 1 member C1	37.4	9.56E-10	4.86	24	0
3	FABP7	O15540	Fatty acid-binding protein, brain	21.9	2.89E-06	4.22	15	0
4	ENOB	P13929	Beta-enolase	22.2	2.50E-06	3.61	18	1
5	AL1A1	P00352	Retinal dehydrogenase 1	11.8	6.07E-04	3.55	9	0
6	4F2	P08195	4F2 cell-surface antigen heavy chain	11.8	6.07E-04	3.55	9	0
7	1C12	P30508	HLA class I histocompatibility antigen, Cw-12 alpha chain precursor	11.8	6.07E-04	3.55	9	0
8	TBA4A	P68366	Tubulin alpha-4A chain	11.8	6.07E-04	3.55	9	0
9	LG3BP	Q08380	Galectin-3-binding protein precursor	20.6	5.77E-06	3.54	17	1
10	1C03	P04222	HLA class I histocompatibility antigen, Cw-3 alpha chain precursor	10.1	1.48E-03	3.40	8	0
11	TKT	P29401	Transketolase	8.46	3.62E-03	3.24	7	0
12	VTNC	P04004	Vitronectin precursor	6.85	8.87E-03	3.05	6	0
13	G6PD	P11413	Glucose-6-phosphate 1-dehydrogenase	6.85	8.87E-03	3.05	6	0
14	PRDX4	Q13162	Peroxiredoxin-4	6.85	8.87E-03	3.05	6	0
15	VDAC1	P21796	Voltage-dependent anion-selective channel protein 1	6.85	8.87E-03	3.05	6	0
16	1B15	P30464	HLA class I histocompatibility antigen, B-15 alpha chain precursor	6.85	8.87E-03	3.05	6	0
17	VILI	P09327	Villin-1	6.85	8.87E-03	3.05	6	0
18	DESP	P15924	Desmoplakin	11.19	8.24E-04	2.96	11	1
19	AHSA1	O95433	Activator of 90 kDa heat shock protein ATPase homolog 1	5.27	2.18E-02	2.84	5	0
20	COPB	P53618	Coatomer subunit beta	5.27	2.18E-02	2.84	5	0
21	TMEDA	P49755	Transmembrane emp24 domain-containing protein 10 precursor	5.27	2.18E-02	2.84	5	0
22	CD44	P16070	CD44 antigen precursor	5.27	2.18E-02	2.84	5	0
23	COPA	P53621	Coatomer subunit alpha	5.27	2.18E-02	2.84	5	0
24	TBB4Q	Q99867	Putative tubulin beta-4q chain	5.27	2.18E-02	2.84	5	0
25	THIL	P24752	Acetyl-CoA acetyltransferase, mitochondrial precursor	5.27	2.18E-02	2.84	5	0
26	EFTU	P49411	Elongation factor Tu, mitochondrial precursor	14.88	1.14E-04	2.47	19	4
27	IDHP	P48735	Isocitrate dehydrogenase [NADP], mitochondrial precursor	13.55	2.32E-04	2.39	18	4
28	LRC47	Q8N1G4	Leucine-rich repeat-containing protein 47	5.41	2.01E-02	2.39	7	1
29	CO6A1	P12109	Collagen alpha-1(VI) chain precursor	4.08	4.34E-02	2.20	6	1
30	PSA	P55786	Puromycin-sensitive aminopeptidase	4.08	4.34E-02	2.20	6	1
31	IMB1	Q14974	Importin subunit beta-1	5.95	1.47E-02	2.17	9	2
32	PSA2	P25787	Proteasome subunit alpha type-2	4.72	2.99E-02	2.02	8	2
33	FAS	P49327	Fatty acid synthase	9.93	1.63E-03	1.93	18	6
34	A1AT	P01009	Alpha-1-antitrypsin precursor	4.05	4.41E-02	1.62	10	4
35	ROA1	P09651	Heterogeneous nuclear ribonucleoprotein A1	6.43	1.12E-02	1.58	16	7
36	FINC	P02751	Fibronectin precursor	8.01	4.64E-03	1.57	20	9
37	TRAP1	Q12931	Heat shock protein 75 kDa, mitochondrial precursor	6.26	1.24E-02	1.50	17	8
38	MYH14	Q7Z406	Myosin-14	6.26	1.24E-02	1.50	17	8
39	ANXA2	P07355	Annexin A2	5.46	1.94E-02	1.49	15	7
40	PHB2	Q99623	Prohibitin-2	4.67	3.07E-02	1.49	13	6
41	GSTP1	P09211	Glutathione S-transferase P	10.63	1.12E-03	1.38	32	17
42	PDIA1	P07237	Protein disulfide-isomerase precursor	8.96	2.76E-03	1.33	29	16
43	1433G	P61981	14-3-3 protein gamma	8.11	4.40E-03	1.28	28	16
44	ACTN4	O43707	Alpha-actinin-4	8.82	2.98E-03	1.26	31	18
45	PCBP2	Q15366	Poly(rC)-binding protein 2	5.03	2.49E-02	1.16	21	13
46	TPIS	P60174	Triosephosphate isomerase	6.45	1.11E-02	1.12	28	18
47	TRFE	P02787	Serotransferrin precursor	7.17	7.41E-03	1.12	31	20
48	ARF1	P84077	ADP-ribosylation factor 1	5.00	2.53E-02	1.09	23	15
49	PCBP1	Q15365	Poly(rC)-binding protein 1	4.31	3.79E-02	1.01	23	16
50	CO6A3	P12111	Collagen alpha-3(VI) chain precursor	5.16	2.31E-02	0.91	32	24
51	EF1A1	P68104	Elongation factor 1-alpha 1	4.72	2.98E-02	0.83	35	28
52	PDIA6	Q15084	Protein disulfide-isomerase A6 precursor	4.01	4.52E-02	0.81	31	25
53	G3P	P04406	Glyceraldehyde-3-phosphate dehydrogenase	14.21	1.64E-04	0.77	113	95
54	ENPL	P14625	Endoplasmin precursor	5.76	1.64E-02	0.66	62	56
55	TBB2A	Q13885	Tubulin beta-2A chain	5.80	1.61E-02	0.63	69	64
56	VIME	P08670	Vimentin	5.92	1.49E-02	0.60	77	73
57	HBB	P68871	Hemoglobin subunit beta	4.88	2.72E-02	-0.53	53	110
58	TBB5	P07437	Tubulin beta chain	10.89	9.64E-04	-0.63	81	179
59	TBA1A	Q71U36	Tubulin alpha-1A chain	14.38	1.49E-04	-0.67	93	211
60	TBB2B	Q9BVA1	Tubulin beta-2B chain	5.93	1.49E-02	-0.69	35	82
61	H2B1B	P33778	Histone H2B type 1-B	6.45	1.11E-02	-0.83	25	65
62	LMNB1	P20700	Lamin-B1	7.27	7.03E-03	-0.87	25	67
63	HBA	P69905	Hemoglobin subunit alpha	5.77	1.63E-02	-0.92	17	48
64	CALM	P62158	Calmodulin	3.90	4.82E-02	-1.00	9	28
65	HNRH1	P31943	Heterogeneous nuclear ribonucleoprotein H	4.36	3.67E-02	-1.01	10	31
66	NUMA1	Q14980	Nuclear mitotic apparatus protein 1	4.83	2.80E-02	-1.01	11	34
67	LAP2A	P42166	Lamina-associated polypeptide 2 isoform alpha	5.31	2.13E-02	-1.05	11	35
68	H31T	Q16695	Histone H3.1t	6.23	1.26E-02	-1.06	13	41
69	GDIA	P31150	Rab GDP dissociation inhibitor alpha	7.65	5.67E-03	-1.09	15	48
70	TBA1C	Q9BQE3	Tubulin alpha-1C chain	14.23	1.62E-04	-1.12	27	86
71	TBA1B	P68363	Tubulin alpha-1B chain	35.27	2.88E-09	-1.16	63	202
72	K1C19	P08727	Keratin, type I cytoskeletal 19	10.64	1.11E-03	-1.19	17	58
73	HSP76	P17066	Heat shock 70 kDa protein 6	6.46	1.10E-02	-1.23	9	33
74	H12	P16403	Histone H1.2	7.59	5.87E-03	-1.31	9	35
75	TBB4	P04350	Tubulin beta-4 chain	12.66	3.73E-04	-1.50	11	48
76	MOES	P26038	Moesin	4.51	3.36E-02	-1.51	3	16
77	KU70	P12956	ATP-dependent DNA helicase 2 subunit 1	22.32	2.31E-06	-1.64	16	75
78	DYHC1	Q14204	Cytoplasmic dynein 1 heavy chain 1	8.54	3.48E-03	-1.67	5	27
79	RBBP4	Q09028	Histone-binding protein RBBP4	6.58	1.03E-02	-1.74	3	19
80	PGS1	P21810	Biglycan precursor	3.99	4.59E-02	-1.81	1	10
81	ROA1L	Q32P51	Heterogeneous nuclear ribonucleoprotein A1-like protein	7.30	6.89E-03	-1.81	3	20
82	HNRPF	P52597	Heterogeneous nuclear ribonucleoprotein F	4.77	2.90E-02	-1.93	1	11
83	RUXG	P62308	Small nuclear ribonucleoprotein G	6.40	1.14E-02	-2.15	1	13
84	1433S	P31947	14-3-3 protein sigma	4.19	4.08E-02	-2.21	0	7
85	PEG10	Q86TG7	Retrotransposon-derived protein PEG10	4.19	4.08E-02	-2.21	0	7
86	CAYP1	Q13938	Calcyphosin	4.19	4.08E-02	-2.21	0	7
87	GBB1	P62873	Guanine nucleotide-binding protein G(I)/G(S)/G(T) subunit beta-1	4.19	4.08E-02	-2.21	0	7
88	NCA11	P13591	Neural cell adhesion molecule 1, 140 kDa isoform precursor	4.19	4.08E-02	-2.21	0	7
89	FSCN1	Q16658	Fascin	5.11	2.38E-02	-2.37	0	8
90	ROA0	Q13151	Heterogeneous nuclear ribonucleoprotein A0	8.98	2.74E-03	-2.43	1	16
91	MDHC	P40925	Malate dehydrogenase, cytoplasmic	7.00	8.13E-03	-2.66	0	10
92	H2A1D	P20671	Histone H2A type 1-D	8.94	2.79E-03	-2.89	0	12
93	SEGN	O76038	Secretagogin	15.915	6.63E-05	-3.51	0	19
94	MAP1B	P46821	Microtubule-associated protein 1B	16.926	3.89E-05	-3.58	0	20
95	BASP	P80723	Brain acid soluble protein 1	24.067	9.30E-07	-3.99	0	27

Proteins are listed in descending order of *R*_*sc *_values, pooled spectral counts are listed, and "_HUMAN" are removed from UniProtKG entry names.

### 3. 3. Biomarker Candidates for LCNEC

**Figure 2:**
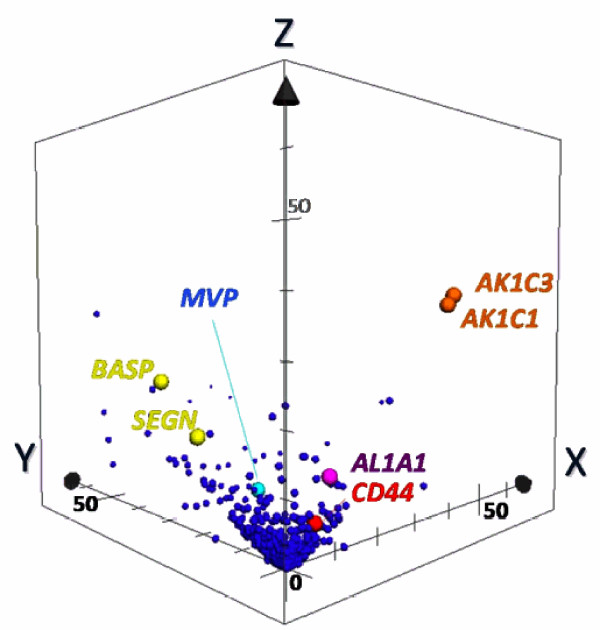
**Marker candidates' extraction by pairwise G statistics**. In the 3D scatter plot, X, Y, Z-axis shows G-values (X: LCNEC vs. LCC; Y: LCC vs. SCLC; Z: LCNEC vs. SCLC). Data point sets from 1,918 proteins were plotted with circles. AK1C1 and AK1C3 (orange), AL1A1 (purple) and CD44 (red) Proteins being located very near or on X-Z plane are isolated as candidates of specific LCNEC markers. SEGN (yellow) were located on Y-Z plane, which was already known as one of SCLC-specific markers.

**Figure 3:**
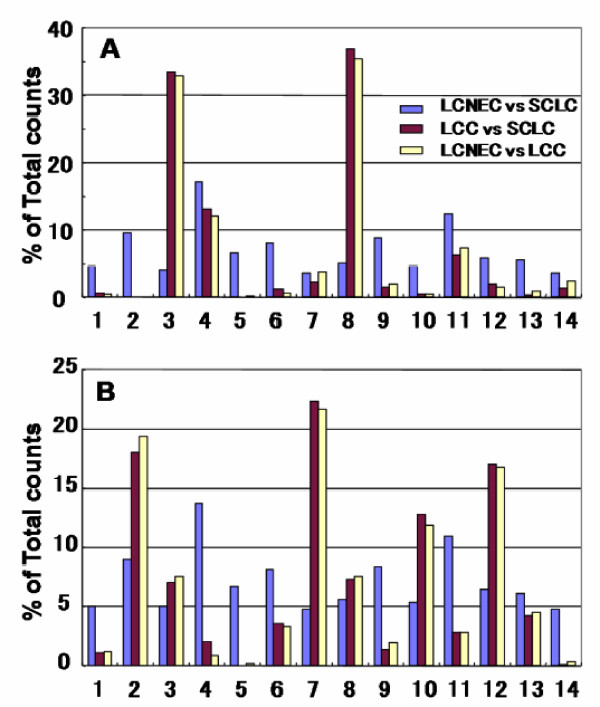
**Gene ontology (GO) analysis on the molecular functions and cellular localization of proteins preferentially expressed in three cancer group pairs (LCNEC vs. SCLC, LCNEC vs. LCC, and LCC vs. SCLC)**. A) Molecular functions: 1, antioxidant activity; 2, auxiliary transport protein activity; 3, binding; 4, catalytic activity; 5, chemoattractant activity; 6, electron carrier activity; 7, enzyme regulator activity; 8, molecular function; 9, molecular transducer activity; 10, motor activity; 11, structural molecule activity; 12, transcription regulator activity; 13, translation regulator activity; 14, transporter activity. B) Cellular localizations: 1, Golgi apparatus; 2, cytoplasm; 3, cytoskeleton; 4, endoplasmic reticulum; 5, endosome; 6, extracellular region; 7, intracellular organelle; 8, membrane; 9, mitochondrion; 10, nucleus; 11, organelle membrane; 12, organelle part; 13, plasma membrane; 14, ribosome.

To illustrate the specificity of protein expression toward LCNEC more clearly, we made a 3D scatter plot with an × axis indicating G-statistic values (*G *values) for LCNEC vs. LCC analysis, a y axis for LCC vs. SCLC, and a z axis for LCNEC vs. SCLC (Figure 2). When the spectral counts of a target protein are zero for both groups in question, it is hereafter defined as *G *= 0. The proteins expressed specifically to LCNEC will therefore be present in the region (x>3.84, z>3.84 corresponding to *p *< 0.05 each) on the x-z plane, those in SCLC in the region (y>3.84, z>3.84) on the y-z plane and those in LCC in the region (x>3.84, y>3.84) on the x-y plane. We used 1,918 proteins for this plotting. Close inspection of the 3D plot shows that AK1C3 at a point (40.8, 0, 39.1), AK1C1 at a point (39.0, 0, 37.4), AL1A1 at a point (8.75, 2.6 × 10^-5^, 11.8) and CD44 antigen precursor (CD44) at a point (5.56, 0, 5.27) are very near or on the x-z plane with convincingly low *p*-values (3.9 × 10^-10^, 9.6 × 10^-10^, 6.1 × 10^-4^, and 0.021, respectively) from LCNEC vs. SCLC comparisons and thus specific to LCNEC. Interestingly, AK1C1, AK1C3, AL1A1, and CD44 have been reported to be biomarkers of cancer stem cells (see Discussion). In Table 2 BASP and SEGN are significantly up-regulated in SCLC compared with LCNEC, which are indeed located on the y-z plane at the respective points (0, 32.2, 24.1) and (0, 21.5, 15.9), and specific to SCLC. Major vault protein (MVP) is at a point (23.8, 34.1, 0) on the x-y plane, indicating an LCC-specific protein. One of well known proteins related to SCLC, γ-enolase (ENOG) is detectable at a point (0.55, 7.23, 2.84) in the 3D G-statistic space which indicates that it is expressed significantly in SCLC compared to in LCC. The G-statistic is assumed to obey a χ2-distribution with one degree of freedom and the *p*-values based on *G*-values obtained with the contingency tables containing small counts should be handled with caution. Therefore we calculated exact *p*-values for the 2 × 2 tables with the non-parametric Fisher's exact test and Mann-Whitney U test. The results were fully consistent with those obtained with the G-test; the exact *p*-values for LCNEC vs. SCLC were 3.40 × 10^-4 ^for AL1A1, 5.53 × 10^-10 ^for AK1C1, 2.27 × 10^-10 ^for AK1C3, and 0.012 for CD44. The G-test analyses of three cancer group pairs (LCNEC vs. SCLC, LCNEC vs. LCC, and LCC vs. SCLC) under *p *< 0.05 retrieved the respective 95, 186 and 237 proteins that showed significant changes in expression levels. These proteins were subjected to gene ontology (GO) analysis, highlighting their biological and molecular functions and cellular localization. As Figure 3 shows, the molecular functions and cellular localization of proteins preferentially expressed in the LCNEC vs. SCLC pair were quite different from those of the other pairs.

### 3. 4. Extended immunohistochemical validation of the proteomics results

**Figure 4:**
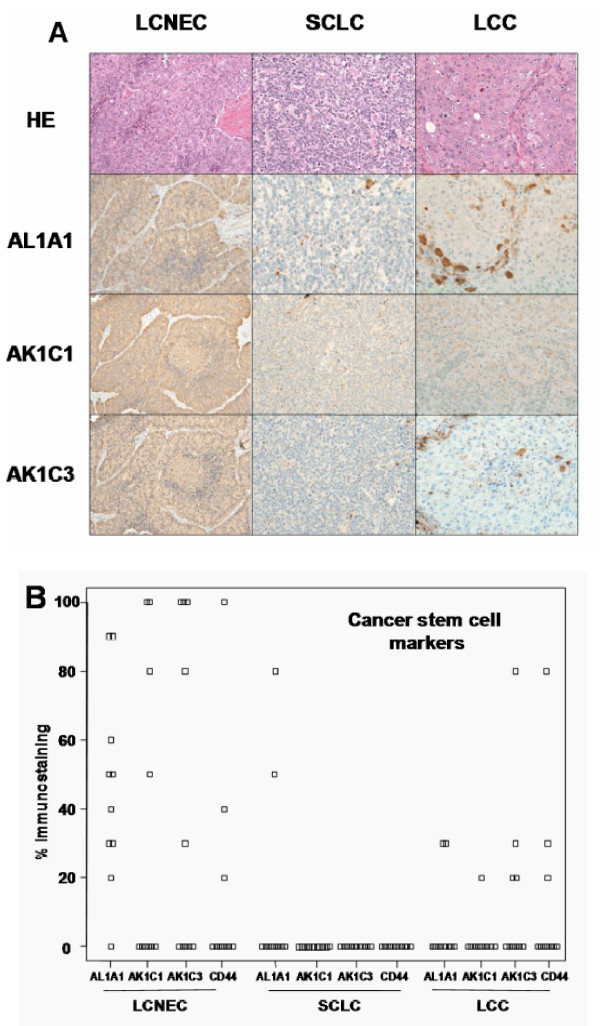
**Imunohistochemical identification of proteomics-identifying proteins**. A) Histological appearances of LCNEC, SCLC and LCC, and immunohistochemical staining of AL1A1, AK1C1 and AK1C3. Magnification, x200. B) Immunoreactivitiy with AL1A1, AK1C1, AK1C3, and CD44. The immunoreactivity was indicated as the percentage of immunopositive area at the maximal cut-surface of tumors.

From this proteomic study we identified AL1A1, AK1C1, AK1C3 and CD44 as biomarker candidates for LCNEC. The results were immunohistochemically verified using a total of 10 cases for each group. We assessed immunoreactivity with the percentage of immunopositive area and staining intensity compared to those of positive-control samples at the maximal cut-surface of tumors (Figure 4). All SCLC cases showed no immunoreactivity with AK1C1, AK1C3 and CD44 and the reactivity of all antibodies with LCNEC sections differed impressively from that of SCLC, supporting the proteomic results. Notably, nine cases of LCNEC including four used for the proteomic experiments were AL1A1 positive in the extent of 30 to 90%. The most intense staining (90% positive area) was observed in patient 2 of LCNEC (Table 1 and Figure 4A). On the other hand, LCC and SCLC sections with typical histology were AL1A1 negative (Figure 4A). There were four cases with weak immunoreactivity (30-80% area) which would contain the small areas mimicking some LCNEC morphology. In LCNEC four were immuno-positive (30-100% positive area) to both AK1C1 and AK1C3, and there was one more AK1C3 positive case. In LCC group one case was AK1C1 positive and four cases were AK1C3 positive; these cases showed small areas with neuroendocrine tendency in the tissue structure. Immnoreactivity of LCNEC cells to CD44 were the same as that of LCC.

## 4. Discussions

This study aimed at developing the way of proteomic distinction between LCNEC and SCLC, which will assist pathologic distinction that has not sometimes been straightforward, leading to therapeutic inefficiency. We have been focusing our attention on using laser-microdissection sampling from FFPE sections for proteomics to explore disease-related protein markers. We have already applied this method to both global semi-quantitative shotgun proteomics using spectral counting and MRM-based quantitative proteomics and successfully identified stage-related proteins on lung AC [11,12]. In this study, we used the same global shotgun method for comparison of three cancer groups (LCNEC, SCLC, and LCC) by spectral counting and explicitly interpreted three sets of pairwise G test results in the 3D G-statistic space (Figure 2). This resulted in identifying four proteins AL1A1, AK1C1 AK1C3 and CD44 that were expressed in LCNEC more than in SCLC and LCC with high probabilities. These proteomic findings using the limited scale of patients were confirmed by routine immunohistochemitry with additional patients. Moreover we identified other proteins related to these cancer groups in the present study, further demonstrating the technical feasibility of this FFPE proteomic method. The identified four proteins physiologically take part in known metabolic processes. AL1A1, AK1C1 and AK1C3 are cytosolic oxidoreductases that are involved in reduction of progesterone to the inactive form 20-alpha-hydroxy-progesterone, metabolism of steroids and prostaglandins with multi-specificity, oxidation of retinal to retinoic acid and the precursor of the storage form vitamin A, respectively. CD44 is one of cell-surface glycoproteins which relates to cell-cell interactions including adhesion and migration, and thus to tumor growth and progression [15]. When we have considered the properties common to these proteins that have apparently no functional relationship with one another, we noticed that AL1A1 [16,17], AK1C1 [18], AK1C3 [19] and CD44 [20] have been proposed to be the markers of cancer stem cells. Their expression in tumor cells could correlate with their aggressive biological behavior, drug resistance and poor prognosis, which are common characteristics of LCNEC and SCLC. The preferential expression of the cancer stem cell markers in LCNEC over SCLC suggests that the mechanism of increasing the extent of malignancy in LCNEC differs from that in SCLC. Previous studies suggested that these redox enzymes were present in a variety of malignant tumor cells. In particular, AK1C1, and AK1C3 are reported in human non-small cell lung carcinoma (A549) cells [21], and a high expression of AL1A1 in lung cancer cell lines, especially in AC cell lines compared to LCC and SCLC cell lines [22-24]. To our knowledge, however, this is the first report of the statistically significant proteomic detection of AL1A1, AK1C1 and AK1C3 in clinical samples of lung cancers, especially in LCNEC. Out of the top five LCNEC-specific proteins, brain-type FABP7 is present in highly infiltrative malignant glioma and associated with enhanced cell migratory activity and thus with poor prognosis [25], suggesting for its involvement in the aggressive nature of LCNEC. Out of the top five down-regulated LCNEC proteins compared with SCLC, BASP is a potential tumor suppressor [26], consistent with its down-regulation in LCNEC, and its specific expression in SCLC suggests that different mechanisms of tumor growth could operate between LCNEC and SCLC. Another SCLC-specific SEGN is a novel neuroendocrine marker that has a distinct expression pattern from the conventional ones used in this study, consistent with being negative in LCNEC, and with the reported rate for positive staining in SCLC (26 out of 31) [27]. The role of AL1A1 in lung cancers is still unknown, but it is recently reported that AL1A1 plays an important role in Notch pathway [28]. Though there has been no effective chemotherapy to LCNEC, Sorafenib, a tyrosine kinase inhibitor in the MAP kinase pathway, is effective to malignant tumor cells with AL1A [29]. AL1A1 would be not only cancer stem cell markers, but also an attractive target of treatment of LCNEC. In addition to statistically sorting protein expression levels by spectral counting, GO mapping of significant proteins on pairwise comparison (*p *< 0.05) provides insights into overall differences from pair and pair in their biological and molecular functions, and cellular components. Gene ontology distributions of molecular function and cellular components in neuroendocrine vs. non-neuroendocrine comparisons, i.e., LCNEC vs. LCC and SCLC vs. LCC, did not significantly differ from each other. On the other hand, those distributions in comparison within neuroendocrine groups, LCNEC vs. SCLC, differed greatly from those of the other pairs. This does encourage us to go ahead with further studies in this line and will promise to get target proteins of LCNEC eventually in future. We checked the rate of positive immuno-reaction of relevant antibodies with proteomics-identifying proteins for ten patients of each group (Figure 4B). Differences between the rates for all target proteins in LCNEC and SCLC are fully consistent with the proteomic results, confirming the specificity to LCNEC. The preferential expression of AL1A1 and AK1C1 in LCNEC over LCC was also immunochemically confirmed, and the rate of AL1A1 positive cases in LCC (20%) agreed with the previous results (25%, 1 of 4) [16]. In contrast, the positive staining rates of AK1C3 and CD44 in LCNEC and LCC were similar to each other. Close inspection of HE sections showed that the positive cases in LCC had small areas with neuroendocrine tendency in the tissue structure as pointed out above. Almost all sections of LCC exhibited no immunoreactivity with the neuroendocrine markers used except for weak reactivity (20 or 30%) in only two cases. This suggests that the LCNEC like structure observed in small portions of LCC sections does not necessarily contain enough secretory granules, but presumably contain LCNEC specific AK1C3 and CD44. Confirmatory conclusion of this issue should await proof by electron micrographic immunohitochemistry. A previous study indicated that CD44 was expressed more in SC (97%) and AC (71%) compared to LCC (29%) and SCLC (0%) [30] in agreement with the present positive rates for LCC (30%) and SCLC (0%).

## 5. Conclusions

We concluded that AL1A1, AK1C1, AK1C3, and CD44 were specific for the LCNEC phenotype in relation to SCLC and LCC through proteomics of FFPE samples. They were useful targets to immunohistochemically distinguish LCNEC from SCLC and LCC. Though we need a variety of studies with more extensive experimental and clinical data to assess the precise function of these marker candidates and confirm them as real biomarkers, this proteomic analysis was effective to detect them and will be applied to other phenotype of malignancies.

## Abbreviations

NSCLC: non-small cell lung carcinoma; LCNEC: large cell neuroendocrine carcinoma; LCC: large cell carcinoma; SCLC: small cell lung carcinoma; CSC: cancer stem cell; LC: liquid chromatography; MS: mass spectrometry; FFPE: formalin-fixed paraffin embedded; LMD: laser microdissection; MS/MS: tandem mass spectrometry; ISIS: in-sample internal standard; AL1A1: aldehyde dehydrogenase 1 family, member A 1; AK1C1: aldo-keto reductase family 1, member C1; AK1C3: aldo-keto reductase family 1, member C3; HE: hematoxylin-eosin

## Competing interests

The authors declare that they have no competing interests.

## Authors' contributions

MN coordinated the clinical and experimental parts of study and drafted the manuscript. TF and KF performed protein analysis through mass spectrometry. TK carried out proteomic data analysis. HT performed statistical analysis and helped to draft the manuscript. MK performed statistical analysis of G-test. YB helped us to use FFPE technique. AG suggested some important points of pathological diagnosis of LCNEC. MT offered clinical samples from patients. HO and TN pathologically diagnosed all samples independently. TO and NI supported us clinically and financially. NG supported us experimentally and financially. HK supported us clinically. GMV and TN coordinated FFPE project and assessed the results. All authors read and approved the final manuscript.
